# Integrated Mechanical, Thermal, Data, and Power Transfer Interfaces for Future Space Robotics

**DOI:** 10.3389/frobt.2018.00064

**Published:** 2018-06-04

**Authors:** Xiu-Tian Yan, Wiebke Brinkmann, Roberto Palazzetti, Craig Melville, Youhua Li, Sebastian Bartsch, Frank Kirchner

**Affiliations:** ^1^SMeSTech Laboratory, DMEM Department, University of Strathclyde, Glasgow, United Kingdom; ^2^Robotics Innovation Center (DFKI-RIC), German Research Center for Artificial Intelligence, Bremen, Germany

**Keywords:** multifunctional interface, robotic space interface, transfer classification in space, modularity, space robotics

## Abstract

*In-situ* connectability among modules of a space system can provide significantly enhanced flexibility, adaptability, and robustness for space exploration and servicing missions. Connection of modules in extra-terrestrial environment is hence a topic of rising importance in modern orbital or planetary missions. As an example, the increasing number of satellites sent to space have introduced a large set of connections of various type, for transferring mechanical loads, data, electrical power and heat from one module to another. This paper provides a comprehensive review of published work in space robotic connections and presents the different transfer types developed and used to date in robotic applications for orbital and extra-terrestrial planetary missions. The aims of this paper are to present a detailed analysis of the state of the art available technologies, to make an analysis of and comparison among different solutions to common problems, to synthesize and identify future connectability research, and to lay the foundation for future European space robotic connectability effort and work for a complex and growing important future space missions. All types are described in their base characteristics and evaluated for orbital and planetary environments. This analysis shows that despite the large number of connectors developed for each of the four functionalities (mechanical, thermal, data, and electrical power) here considered, the trend is that researchers are integrating more than one functionalizes into a single equipment or device, to reduce costs and improve standardization. The outcomes of this literature review have contributed toward the design of a future multifunctional, standard and scalable interface at the early stage of the Standard Interface for Robotic Manipulation of Payloads in Future Space Missions (SIROM) project, a European Commission funded Horizon 2020 project. SIROM interfaces will be employed by European prime contractors in future extra-terrestrial missions.

## 1. Introduction

Modularity in space is one of the main issue that engineers and space mission planner have to face nowadays. With the increasing complexity of mission's plans and tasks, a need for a standard, multifunctional, scalable, and modular interface arise. Future space robotic mission need a set of integrated and inherently optimized interfaces for mechanical, data, electrical, and thermal connectivity that allow reliable, robust and multi-functional coupling of payload to robot manipulators, payload to other payload or client to server, in both orbital and planetary environments.

The present paper reports an overview of classifications of power, data and mechanical interfaces, and thermal transfer methods for space applications. For all types, the main characteristics and functionalities are described and evaluated for orbital and planetary suitability. While in-orbit systems have to operate in harsh, but steady environment, planetary systems deal with potentially highly variable environment with, among others, changing temperature and dust level. A comparison among existing interfaces in robotics and space is also presented, with respect to pre-defined mission scenario and requirements. The main focus is on the different transfer possibilities for space and planetary use, in order to present a clear overview of the state of the art of the various types of functional connectivity.

Existing interfaces are presented in section 2, an outline of each type of transfer is given in section 3, and section 4 presents possibilities and ideas of innovation and development of standard multi-functional interfaces with the most promising developments expected in the next few years. Eventually, conclusions and outlooks are given in section 5.

## 2. Existing interfaces

Terrestrial examples of robotic connection interfaces are commonly seen in modular robotic systems, where homogeneous or heterogeneous modules interconnect to satisfy some functions, either mechanical, thermal, electrical, or computational. Modular platforms make the creation and the operation of large structures easy and also reduce manufacturing and maintenance costs, this is why they are being considered for large scale space operations. A number of space-relevant module interfaces have been found in literature, Table 1 summarizes some selected existing interfaces. The interfaces selected were based on the presence of some key properties: They should have an androgynous docking system and a rigid connection between interfaces through mechanical latching, have power, data or thermal transfer functionalities, have a form of redundancy or be capable of angular orientation.

**Table 1:** Basic properties of existing interfaces.

	**Androgynous**	**Rigid connection**	**Mechanical**	**Latch type**	**Power**	**Data**	**Thermal**	**Redundancy**	**Marker**	**Angular orientation**	**No. of orientations**
MTRAN				Magnetic	X	X				X	4
SINGO	X	X	X	Clamp				X		X	4
CAST		X	X	Hook						X	2
CEBOT			X	Hook					X		1
ATRON		X	X	Hook		X					1
Telecube	X			Magnetic	X	X		X		X	2
PolyBot	X	X	X	Rotational	X			X		X	4
AMAS		X	X	Hook				X		X	4
SMORES	X			Magnetic						X	2
GENFA	X	X	X	Rotational	X			X		X	4
ACOR			X	Hook	X			X			1
SWARM	X	X	X	Rotational	X			X			1
Phoenix Tool		X	X	Hook	X			X		X	1
Phoenix Satlet		X	X	Clamp	X	X		X		X	1
SSRMS LEE		X	X	Snare	X	X		X			1
DEXTRE (OTCM)		X	X	Clamp	X	X					1
iSSI	X		X	Rotational	X	X	X	X	X	X	4
EMI		X	X	Clamp	X	X		X	X	X	4
Berthing and Docking		X	X	Hook				X		X	∞
EM-Cube	X			Magnetic				X	X	X	2

The main purpose of an interface in this context is to establish a rigid connection and transfer resources of various type (e.g., mechanical, electrical, data, thermal) between two units. Interfaces which only transfer mechanical force to dock two robotic systems with each other are perhaps the most basic and most common in simple module systems. Most of these interfaces exist to demonstrate latching methods between modules. These examples are SINGO (Shen et al., 2009) with an androgynous clamp latch type, the Compliant-And-Self-Tightening (CAST) system (Khoshnevis et al., 2003) with a hook mechanism, the Cellular Robotic system (CEBOT) (Fukuda et al., 1989) with a peg-in-hole physical guidance system and hook latch mechanism, the Automatic Modular Assembly System (AMAS) (Sproewitz et al., 2008) with a hook mechanism, as well as the Berthing and Docking Mechanism of DEOS mission (Rank et al., 2011), and the semiandrogynous docking mechanisms (Olivieri and Francesconi, 2016). The mentioned systems are modular systems and have mechanical mechanism, which ensure a rigid connection once the robotic systems are docked with each other, highly important for robots containing modules with locomotive sub-functions. EM-Cube also is an interface with one mechanical connection type, but uses electromagnets and permanent magnets to enable the cubes to slide along their surface and to dock cubes with each other (An, 2008). The Self-assembling Modular Robot for Extreme Shapeshifting (SMORES) uses a magnetic connection by using permanent magnets (with 180° symmetry) and a docking key (used to reinforce a connection or undock) (Davey et al., 2012). These magnetic interfaces are non-mechanical and are considered semi-rigid. While under very small amounts of force, powerful neodymium magnets locked together will not exhibit any translational movement, however a stronger shearing force will unlock the magnets, meaning the interface is subject to breaking under nominal conditions.

Interfaces that are designed to transfer power mostly have a mechanical latching system and ensure electrical power transfer; examples are PolyBot (Yim et al., 2000), GENFA (Fu et al., 2011), ACOR (Badescu and Mavroidis, 2003), SWARM (Rodgers et al., 2005), and the tool changer system used in the DARPA's Phoenix project (Phoenix Tool) (Henshaw, 2010). Whereas, ATRON uses three point-to-point hooks as well as four IR emitters and receivers as a rudimentary distance sensor that also transfers information between neighboring modules (Jørgensen et al., 2004), MTRAN (Murata et al., 2002), and Telecube (Suh et al., 2002) are exceptions to the mechanical latch rule and use magnetic latches for modules that transfer both power and data.

The Phoenix Satlet interface (Henshaw, 2010), the Space Station Remote Manipulator System (SSRMS) with the Latching End Effector (LEE) (Lee et al., 1995), the Orbital Replacement Unit Tool Change-out Mechanism (OTCM) of the DEXTRE robot (Hwang, 2013), and the electromechanical interface (EMI) (Wenzel et al., 2015) enable mechanical connection, power transmission, and data transfer. In addition, OTMC and the EMI use a video/camera for viewing the docking procedure. These interfaces are considerably more complex than the single resource type of module interface, due to their scale and compounding set of requirements. These designs in particular are part of an industry organized and well-funded effort to produce flight-ready designs, and so the complexity and refinement in the designers are very pronounced.

Currently the interface of intelligent Satellite System interface (iSSI) (Goeller et al., 2016) is the only design that enables mechanic, thermal, power, and data transfer functionalities.

Rotational orientation of an interface facilitates the docking procedure, for future space and planetary missions it is a useful attribute to have at least 4 docking orientations, such as in MTRAN, SINGO, PolyBot, AMAS, GENFA, iSSI, and the EMI. These designs allow for minimal adjustments to alignment during docking, particularly rotational alignment, and thus reduce fuel consumption and failure modes. Rotational symmetry also helps incorporate redundancy of interface components.

Redundancy of a system is a key feature for space and planetary missions as it increases the system's tolerance to component failures. As soon as a (robotic) system leaves the Earth and therefore out of reach of humans it must be able to cope with failures without human assistance since maintenance is not an option. This means if a part of a system fails, there should be at least one facility to compensate the failure. SINGO, Telecube, PolyBot, AMAS, GENFA, ACOR, SWARM, Phoenix Tool, Phoenix Satlet, SSRMS LEE, iSSI, EMI, the Berthing and Docking Mechanism and the EM-Cube are all interfaces with redundancy elements.

It can be concluded that there are a series of beneficial traits that designed interfaces have in common which match the ideal design traits: it can also be verified that rotational symmetry, redundancy, rigid connectivity, and androgyny are understood in the field to be desirable traits for module interfaces.

## 3. Classifications

This section describes and compare the main techniques used for mechanical connection and electrical, data and thermal transfer. Tables in each section present an overview of the advantages and disadvantages of each type.

### 3.1. Mechanical

**Figure 1:**
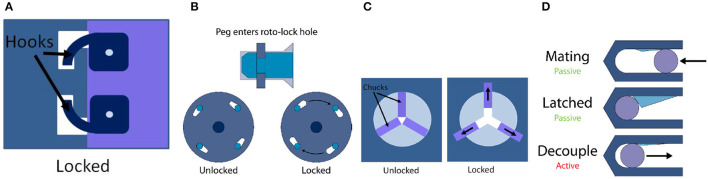
Latching mechanism. **(A)** Hook; **(B)** Roto-Lock; **(C)** Clamp; **(D)** Carabiner.

In this section the mechanisms used to physically secure and connect two modules are presented. During the years, hundreds of different mechanical connection designs have been developed for various purposes, and listing them all would far exceed the limits of this paper. However, most of them fall into four main types: hook, rotational, clamp, and carabiner. The main characteristics of each are presented in Figure 1.

Latches are used to lock or restrict movement after the initial contact; they can be activated automatically once the connectors have been firmly pushed together or engaged. Sometimes latches are not necessary, but they are strongly recommended to ensure rigid connection. Latching can be achieved mechanically or magnetically, and are often motor driven, but can also be initiated in other ways [e.g., by Shaped Memory Alloys (SMAs), despite the high current they require to be activated; Khoshnevis et al., 2003]. Table 2 summarizes advantages and disadvantages of the four mechanical latch classes.

**Table 2:** Advantages and disadvantages of mechanical latch classes.

	**Advantages**	**Disadvantages**
Hook	Rigid connection	Naturally male
	Small misalignment correction	Multiple moving parts
	Passive retention	Point contacts
		Active couple/decouple
Roto-Lock	Rigid connection	Little/no lateral misalignment correction Active couple/decouple
	One moving part	
	Passive retention	
	Potentially fail-safe	
Clamp	Naturally male or female	Multiple moving parts
	Rigid connection	Active couple/decouple
	Fail-safe	
	High strength	
	Passive retention	
	High misalignment correction	
Carabiner	High misalignment correction	Naturally female
	Passive couple	Multiple moving parts
	One moving part	May require push force
		Active decouple

#### 3.1.1. Hook

The hook latch is perhaps the simplest design of them all: one side of the connector rotates hook-like appendages into position around the other face of the connector, interfering in any translational movement perpendicular to the face, as well as rotational movement around that axis (see Figure 1A). The locking mechanism can be either passive (using e.g., springs) or active (using actuators).

A typical hook connector has been presented by Jørgensen et al. (2004), where hook-like connectors are employed to engage identical modules, actuated by DC motors. In the design proposed by Khoshnevis et al. (2003), instead, the actuator is powered by a SMA wire that secure the female part of the hook to the male one. A similar approach has been used by Badescu and his team (Badescu and Mavroidis, 2003), that uses SMA to actuate circular series of lamellae in a clever self-locking mechanism.

#### 3.1.2. Roto-lock

A rotational locking mechanism is a motor-powered type of lock, that requires a male/female interface to operate. It usually works by first having the male side of the connection coupled with the female counterpart; when the roto-lock engages, rotating around the center of interface, it tightens the female side or latches into groves on the male side (see Figure 1B). This concept is almost exclusively used with the Peg-in-hole system, and its main benefits are that it only involves one moving part, highly beneficial for space applications, that it can be developed in a genderless configuration (suitable for coupling identical interfaces), like in the iBoss project (Kortman et al., 2015), and that it is usually smaller compared to other configurations. The Phoenix Tool program (Henshaw, 2010) presents a different design on the similar principle, where locking balls are used to keep the two parts of the interface together.

#### 3.1.3. Clamp

**Figure 2:**
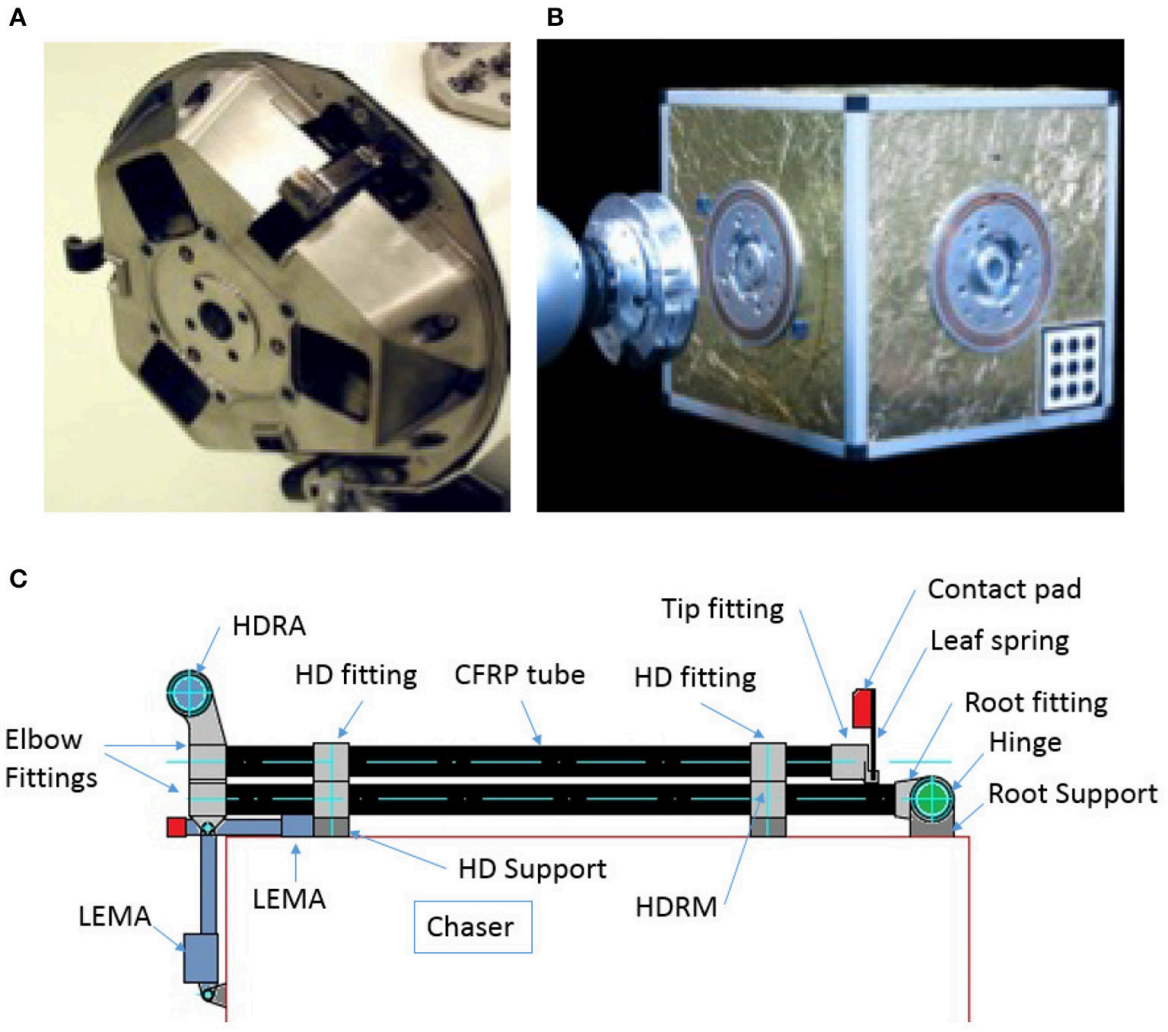
**(A)** Three clamps in a grapple mechanism of ERA (Source: Cruijssen et al., 2014) [Reproduced with permission of the lead author and Dutch Space BV, Leiden, The Netherlands]; **(B)** A robotic docking system of mechanical interface (Source: Kortman et al., 2015) [Reproduced with permission of FZI Research Center for Information Technology, Karlsruhe]; **(C)** clamping in a grapple mechanism design (Source: Meyer et al., 2015) [Reproduced with permission of SENER Ingeneria y Sistemas S.A., Spain and ESTEC ESA, Noordwijk, The Netherlands].

The clamp mechanism involves two or more chucks (jaw-like contraptions) moving radially together, that connect/disconnects the two interfaces, as shown in Figure 1C (a three-chuck clam). Such an example with three clamps shown in Figure 2 can be found in the grapple mechanism of the European Robotic Arm (ERA).

In a typical male-female interface arrangement, the clamp may be on the female side, but there are ways to produce a hermaphroditic clamp connection, where the clamps are the main points of contact on both faces. Regular chucks are simply holding the rod as a push-fit solution, and with enough force, Z axis and rotation can happen; it can be avoided by using modified notches in the chucks and male pieces.

Figure 2B shows a design and prototype of an integrated mechanical interface entitled iBOSS for satellite interfacing with a potential for robotic interface (Kortman et al., 2015).

A conceptual design of a clamping mechanism for a grapple system for active space debris removal based on tentacles, has been developed and matured to a level with development and verification plan. Tentacles are composed of a double boom allocated at a chaser satellite's middle plane and the tentacle is clamped by a hook device driven by a Linear Electro-Magnetic Actuator. The concept is developed from a trade-off of several concepts, including seven tentacles based concepts, one linear stroke clamp and a boom on a four link mechanism (Meyer et al., 2015).

The main advantage of this configuration is that it enables self-aligning, which is of fundamental importance for a large number of applications, and where additional connectors, requiring precise alignment, are involved.

#### 3.1.4. Carabiner

This latch is made of a mechanical interference piece in a passive locked state on the female side, and one crossbar piece on the male part (see Figure 1D). The male part pushes into the female one to disengage the interference piece using a small amount of force. Once the male part is past a certain threshold, the female piece returns to its default position either through active (with an actuator or an electromagnet) or passive (by a spring or a static magnet) means. The lock can only be disengaged using an active unlocking sequence, but the translational motion offers many options for automatic alignment.

### 3.2. Electrical

**Figure 3:**
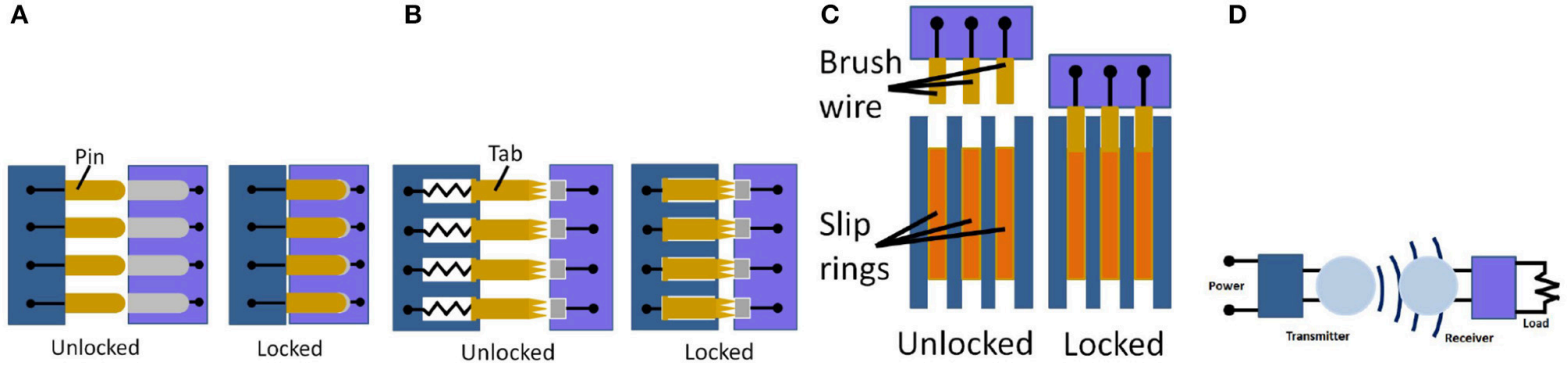
Electrical transfer types. **(A)** Pins; **(B)** Tabs; **(C)** Slip Rings; **(D)** WPT.

The transfer of electrical power between extra-terrestrial modules follows a similar approach to that on Earth, but the cold vacuum of space adds some challenges to the process. In particular, the wide temperature range makes on-purpose space-designed cables necessary. The power transfer is a common function for modern and dated interfaces. Four main designs have been developed for the purpose and here described: pin, tabs, slip rings, and wireless power transmission, as shown in Figure 3.

Advantages and disadvantages of each of these types are presented in Table 3.

**Table 3:** Advantages and disadvantages of electrical connections.

	**Advantages**	**Disadvantages**
Pin	Prevent lateral movement	Easily breakable if thin
	Large electrical connection surface	Affected adversely by particles
	Latching after connection	Needs precise guiding
	Transfer of energy and signals	Sizes dependent on power loads
Tab	Large angular/displacement	No latching after connection
	tolerance	Sizes dependent on environment
	Power available before latch fully closed	
	Tolerance against particles	
	Transfer of energy and signals	
Slip rings	Applicable for high power	Wear of sliding contact
	Transfer of energy and signals	High assembly space
	High torque at start	Unsuitable for short term
	Low starting current	operation
	High over loading capacity	Long initial phase
	Rotating connection	
Wireless	Non-contact transmitter receiver	Large surface for energy
	Resistant against affecting by	absorption
	particles	Weight penalty
	Insensitive to interference	Loss of energy
	Good stability	

#### 3.2.1. Pins

Pins are a versatile way of interlocking and maintaining electrical contact between two interfaces. It involves the use of male pins and female inserts (see Figure 3A), usually quite long and cylindrical. The connections are made between a pin and a hole insert through multiple contacts between the pin and its typical circular insert with very little adjustable hole diameter. There is often a force required to push the pins into the insert to ensure a good contact. This type of connection prevents lateral movement, while axial translation is still allowed without a dedicated latching mechanism. The arrangement of pins determines redundancy, rotational symmetry, and gender of the interface. Pins are affected adversely by particles. This is due to their reliance of good contacts enabled by strict geometric design and manufacture of these pins and their mating hole inserts, as unlike tabs and even slip rings, there is no or little self-adjustment capability enabled in designs. Particles can also cause increased wear and tear on pins and holes which can result in unreliable connections. Depending on the sizes of the pin's diameters and materials, pins may be easy to be bent or even break during connecting or once disconnected and exposed to make contact with external objects, causing unreliable interfacing problems in connection.

**Figure 4:**
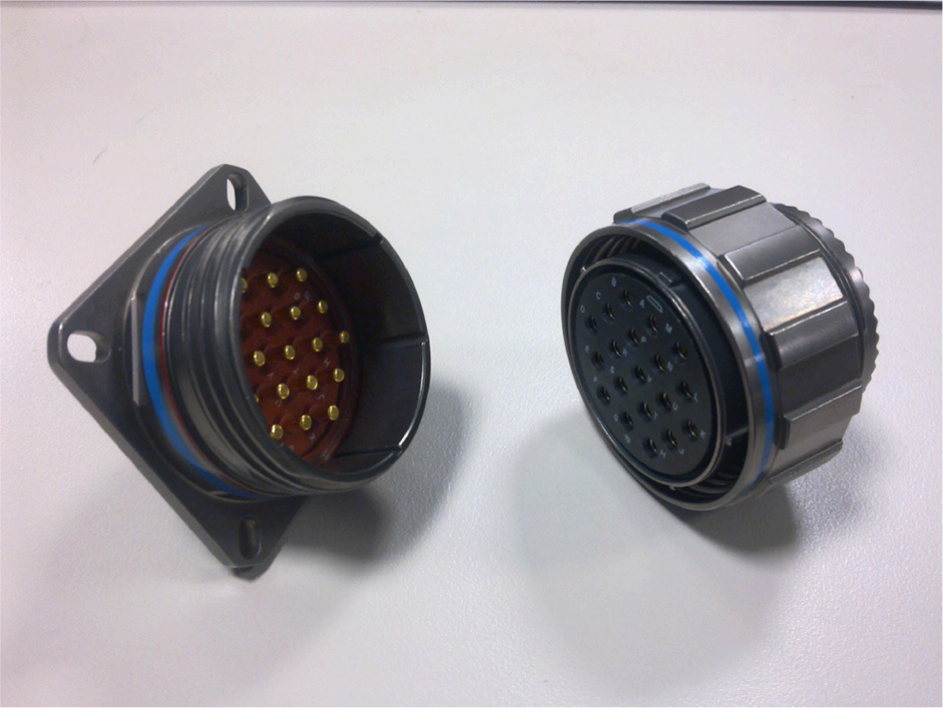
Example of a MIL-DTL-38999 pin connector (photo: https://commons.wikimedia.org/wiki/File:38999_connector_nickel-teflon.jpg, Accessed 03/05/18, Abaillieul. 2010. CC BY-SA 3.0).

Figure 4 shows a real electrical connector with pins.

#### 3.2.2. Tabs

Tab contacts (see Figure 3B) are spring loaded metal component that act as simple touch interface for power. They are not designed with any form of latching, but the spring load gives the interface excellent angular and axial tolerance, enabling the connection even before the latch has fully closed. As a result, tabs possess reliable connection due to its self-adjustment of tabs axial movements enabled by springs to allow a good contact between within a tab. This capability will enable it to withstand the abrasion caused by external particles. Tabs need to be sized carefully to compensate for high power loads and other space environment effects. This is due to the use of springs which has different cross-section possibly different materials to the connecting pins, hence different current conducting abilities.

**Figure 5:**
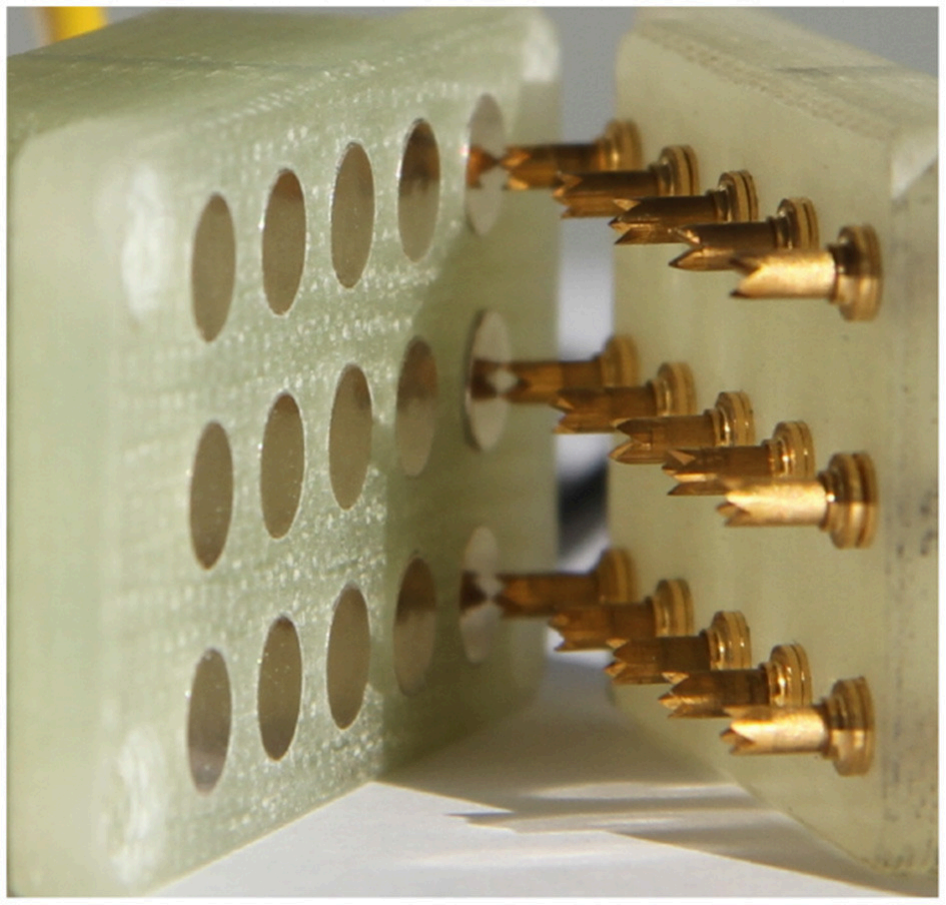
Real tab contacts (Reproduced with permission of: DFKI GmbH).

Figure 5 shows a Spring-loaded Tabs used on the EMI (Wenzel et al., 2015) and in the Phoenix Satlet interface project.

Often these power connectors are equipped with the long scoop-proof power shells, preventing inadvertent cocking of the mating plug connector into the receptacle. Such cocking can occur in other connector designs, causing physical damage or electrical shorting. In the event of connector mismatches, scoop-proofing will also preclude the inadvertent partial electrical mating of differently keyed connectors.

#### 3.2.3. Slip rings

Slip rings are electrical contacts in ring form, allowing for theoretically infinite number of rotational allowances (see Figure 3C). Slip rings have traditionally been used to transfer power across these rotating interfaces. They demand much more space than other methods of power conduits, but provide a more flexible solution for abstract rotation connections. They also suffer a short time operating due to mechanical contacts and wear. Regular maintenance service is hence required.

**Figure 6:**
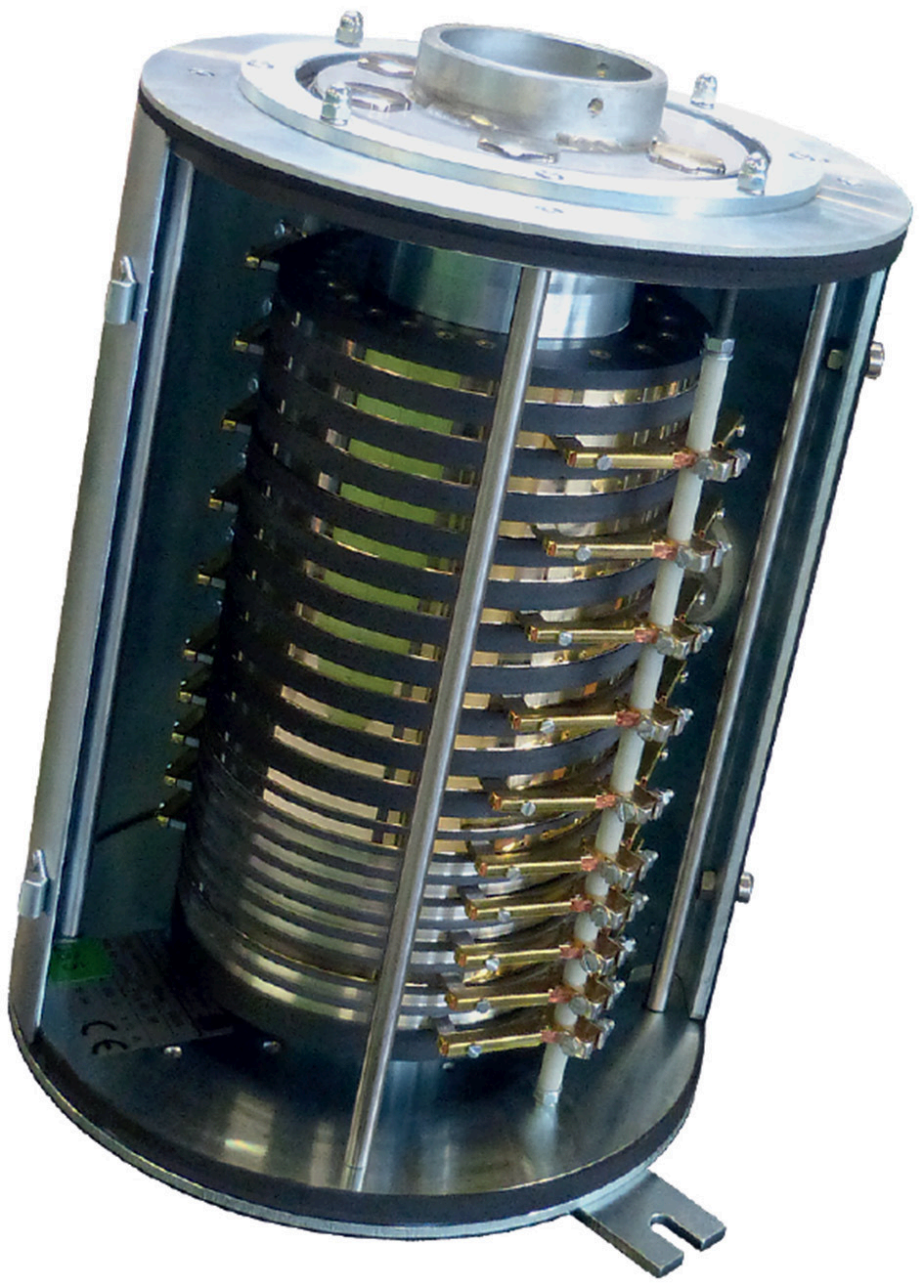
A cast slip ring assembly with carbon brush system for data transmission [Reproduced with permission of Wabtec (Faiveley, Stemmann-Technik GmbH, Schüttorf)].

They have also been used to transmit data and control signals with an increasingly high speed over either copper connector or fiber connector, as shown in Figure 6 and another example of power and signal transmission can be found in Dorsey (2005). In addition, vibrations in any use scenarios will cause the spring in a slip ring contact structure to move axially, resulting in less reliable contact performance. This is a key reason why slip ring contacts are not widely used in space applications.

#### 3.2.4. Wireless power transmission

Wireless power transmission (Dankanich et al., 2015) is a high specific power electric propulsion enabled by disassociating the power generation from the transfer vehicle. First, DC power is acquired on the power-beamers through the use of solar arrays, and then converted to RF power so that it may be transmitted to the rectenna (rectifying antenna) on the transfer vehicle. A rectenna captures incident RF power and transforms it in DC power again by a diode based converter (see Figure 3D). This system increases, by one order of magnitude, the actual specific power transferred to spacecrafts.

**Figure 7:**
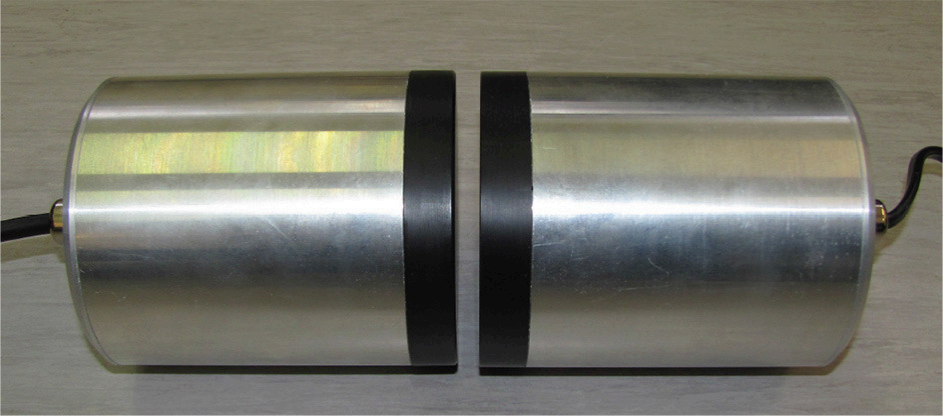
Wireless power transmission components Rotenda M of the company KONTENDA (Reproduced with permission of: DFKI GmbH).

Figure 7 shows real components for wireless power transmission used by the company KONTENDA.

A quick look at typical spacecraft power bus types can give a clear picture of what kind of standards to expect when designing for power transfer. Table 4 shows the typical craft bus voltage ranges and some example craft.

**Table 4:** Typical spacecraft bus voltages.

**Bus voltage (V)**	**Example spacecraft**	**Typical power (kW)**
28	Early sats.	≤2
50	BSS601	1–5
70	LM A2100	2–10
100	LM7000, BSS702	5–20
120	ISS	10–100
160	ISS	10–100
200	SP-100	20–1,000

While 120–160 V is the operating range of ISS modules, 100 V is a current trend in more modern satellite systems. This standard is utilized by large system integrators such as Lockheed Martin and Boeing (MacDonald and Badescu, 2014).

### 3.3. Data

On-board bus requirements are currently driven by the need to move from fully centralized processing toward distributed processing. A modern spacecraft bus needs to be able to acquire synchronous data frames from sensors with controlled latency, transmit synchronous to actuators with controlled latency, transfer asynchronous and isochronous data packets between nodes, and provide a symmetric medium access control service to nodes (i.e., each node can access the bus on demand), accurate distribution of time data and time reference pulse, and a safe implementation for a cross-strapping mechanism (Stakem, 2014).

Many industry-standard serial data communications methods are in use in space. The limiting factor is often the availability of space-qualified supporting hardware for a given bus, though software protocols may remain the same. Although I2C, SPI, PCI, and even USB have been long tested and used as board-level data buses in space, with appropriate redundancy and software support, a Modular Data Interface is expected to use a communications bus suitable for reliable long-range wired links.

The performance of data transmission depends on the maximum length of copper transmission line that can support the chosen data format ranging from 10 BASE2 to 1,000 BASE-CX or IEEE-1394b with a data transmission rate of 800 Mbps as long as the desired data rate doesn't exceed the bandwidth limit of the copper transmission line. As an example, for 1,000 BASE-CX, the maximum transmission line length is 25 m.

Eight main data buses have been highlighted from literature and are here briefly presented. Advantages and disadvantages of these data transfer types are shown in Table 5.

**Table 5:** Advantages and disadvantages of data transfer types.

	**Advantages**	**Disadvantages**
MIL-STD-1553b	Considerable flight heritage	No remote control when
	Deterministic behavior	data is transmitted
	Established spacecraft	Bus central control by Bus
	standards	Controller node
	Galvanic isolation of nodes.	Max. data rate of 1 Mbit/s
	Provision for redundant bus.	
CANbus	ECSS standard for CANopen	Max. data rate of 3.7 Mbit/s
	Large terrestrial knowledge base	
	Very low resource requirements	
	Ability to meet deterministic timing constraints	
	In-built error capabilities	
SpaceWire	Established flight heritage	Multiple competing
	Developed specifically for space	SpaceWire protocols
	environment	Hardware can be expensive
	Capable of large data rates	
	Capable of deterministic behavior	
	Flexible, scalable and robust	
SSI	Differential Signaling	Max. data rate 10 Mbit/s
	Good noise immunity	No higher level networking features
	Capable of deterministic behavior	
Time-Triggered Bus	Deterministic behavior	No flight heritage
	High data rates	Limited use
	Reliable and robust	Small number of component suppliers
Firewire	High data rates	Limited flight heritage
	Military and Aerospace Firewire standard	Limited cable length
	8b10b encoding	
	Low Voltage Differential Signaling	
	Deterministic behavior	
TTE	Ability to send time/event	Very limited flight heritage
	triggered messages	No ECSS standard yet
	Flexible and scalable network topologies	
	High data rates	
	Deterministic behavior	

#### 3.3.1. Milbus

One of the first military-spec data buses was the Mil-Std-1553B Manchester code bus (Milbus), adopted by the ESA in the ECSS-E-50-13C standard (Notebaert et al., 2009). It is time-division multiplexed, very robust, and has been used in many space applications being self-clocking and capable of detecting many types of communications error. However, the use of Manchester encoding introduces frequency related issues at high data rates, and it may not be suitable for operation at 100 Mbit/s. The MIL-STD-1773 specification improves data rate by using fiber optic media.

#### 3.3.2. CANbus

The Controller Area Network developed by Robert Bosch GmbH is a very popular and common message-based half-duplex bus in robotics and automotive applications, and has been used in several space applications including the original Robonaut (Ambrose et al., 2004). While CAN was originally specified as a link layer only, the ISO11898-2 standard provides definition for a standardized electrical solution. Multiple access to the bus is implicitly allowed such as with open-drain transmitters and terminating resistors, although this limits the speed on a traditional CAN bus to 1 Mbps or lower if longer than 40 m, and supporting higher bit rates on more flexible networks is the focus of the new CAN FD Standard (Hartwich and Bosch, 2012). ESA is currently fostering the adoption of the ECSS CAN standard ECSS-E-ST-50-15 which includes the CANopen protocol for synchronous data transfers, frame identifiers, and redundancy management.

#### 3.3.3. SpaceWire

One of the newest and most space-centered buses is the SpaceWire, which focuses on connecting processing nodes via reliable full-duplex switched serial packet links (Parkes, 2012). The SpaceWire communications standard ECSS-E-ST-50-12C has been supplemented by protocol identification in ECSS-E-ST-50-51C, remote memory access in ECSS-E-ST-50-52C, and packet transfer in ECSS-E-ST-50-53C, and adopted by ESA, NASA, JAXA, and RosCosmos (Roberts and Parkes, 2010). As a decentralized network, it is well- suited to redundancy and multi-node robotic systems and is much simpler and more reliable than traditional spacecraft backplanes. SpaceWire is typically limited by hardware design to 400 Mbit/s, but the underlying LVDS standard can perform much faster with up to 3 Gbit/s possible in concept on terrestrial hardware.

However, support for this standard is still exclusive to space hardware and less common than the buses above.

#### 3.3.4. Standardized serial interface

The RS-422/423 ANSI standards were created as industrial serial bus standards, and have proved vastly superior performance than the previous RS-232 due to the use of lower voltages and differential signaling for higher bit rates. The RS-485 also defines an enhanced RS-422 standard that enables very flexible multiple-point networking options in both half- and full-duplex configurations on a differential bus.

#### 3.3.5. Time-triggered bus

Four time-triggered common bus architectures are the SAFEbus, TTA (Time-Triggered Architecture), SPIDER (Scalable Processor-Independent Design for Electromagnetic Resilience), and FlexRay (Rushby and Miner, 2003). SAFEbus interfaces (Bus Interface Units, BIUs) are duplicated, and the interconnect bus is quad-redundant. Its data rate is limited to 60 MB/s. SAFEbus is the most mature of the four, but also the most expensive.

Commercial development of the TTA architecture is undertaken by TTTech and it is being deployed for safety-critical applications in cars and for flight-critical functions in aircraft and aircraft engines. TTA is unique in being used for aircraft, where a mature tradition of design and certification for flight-critical electronics provides strong scrutiny of arguments for safety. SPIDER is a research platform dedicated to explore recovery strategies for radiation-induced high-intensity radiated fields/electromagnetic interference (HIRFEMI) faults, and the interconnect is composed of active elements called Redundancy Management Units (RMUs). SPIDER uses a different topology and a different class of algorithms from the other three types of buses.

FlexRay, is intended for powertrain and chassis control in cars, and its operation is divided between time-triggered and event-triggered activities. It is interesting because of its mixture of time- and event-triggered operation, and potentially important because of the industrial clout of its developers.

#### 3.3.6. Firewire

Among the major contender for data buses, there are the Firewire (IEEE1394) and the Time-Triggered (TT) Ethernet. Bus IEEE1394 has been firstly introduced in 1995 for real-time high-speed data transmission, and has recently been updated to a real-time standard satisfying space and military avionics interconnect needs (Baltazar and Chapelle, 2001). It is a high versatile system, because of its variable channel sizes, bandwidth on demand, hierarchical addressing, and the 1,600 Mbps data rate with a 64-bit wide data path.

#### 3.3.7. Time triggered ethernet

TT Ethernet is intended to support all types of applications, from simple data acquisition, to multimedia systems up to the most demanding safety-critical real-time systems which require a fault-tolerant communication service that must be certified (Kopetz et al., 2005). It distinguishes between two traffic categories: the time-triggered traffic, that is temporally guaranteed, and the standard event-triggered Ethernet traffic which is handled in conformance with the existing Ethernet standards.

The design of TT Ethernet has been driven by the requirement of certification of safety-critical configurations with respect to the integration of legacy applications and legacy Ethernet hardware.

### 3.4. Thermal

**Figure 8:**
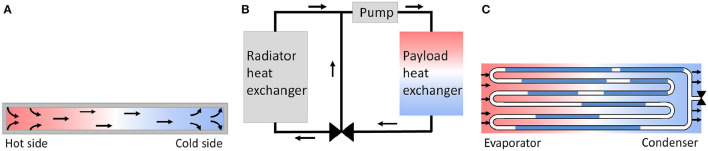
Thermal transfer types. **(A)** Heat pipe; **(B)** MPFL; **(C)** Pulsating heat pipe.

Spacecrafts and satellites in space are exposed to extreme temperatures. Also technical systems, e.g., robotic systems, on different planets within our Solar system work in extremely temperature conditions. While a satellite/spacecraft can be on the cold and hot side of the space at the same time, technical systems on planets undergo in general temperature fluctuation over the time. In order to enable heat exchanges within a technical system, like spacecraft, satellite, or robotic system, or between two connected systems, thermal transfer components will be designed, chosen, and used depending of the required heat transfer and area where the technical system works. Six thermal transfer methods have been identified in literature, and they are here presented. Figure 8 shows the three main methods, and Table 6 presents advantages and disadvantages of each one.

**Table 6:** Advantages and disadvantages of thermal transfer types.

	**Advantages**	**Disadvantages**
Heat pipes	Great efficiency in transferring heat	Not feasible for long distances
	The most reliable	
	Fewer components	
	Well suited for small assembly space	
	Well-founded experiences	
Fluid loops	Large amount of heat at long distance	Leakage
		Large number of components
Water sublimators	Ideal for space	Sensitive
		Large dimension
		Unsuitable for planetary environment
Pulsating heat pipes	Open and closed loop configs	Less experiences
		Few suppliers
Self-rewetting Fluids	Thermal resistance and dry out limit	
	Supplement to heat pipes	
Hybrid systems	Highly variable for different	Large number of
	temperature conditions	components

It should be noted that, while a wide range of thermal transfer methods are available, the literature review has not produced any evidence of a proper thermal interface, intended as a thermal connector between two separate modules. A wide range of thermal transfer methods has been shown, but no one of them can be considered a proper interface. The only relevant interface has been found in Kortman et al. (2015), and it consists of a special carbo-nanotube copper-alloy composite material. This composite features an extremely high conductive heat transfer coefficient which allows for a significant heat exchange between modules even with relatively low contact pressure.

#### 3.4.1. Heat pipes

Loop heat pipes (LHPs) are among the most common thermal transfer methods used in spacecraft. They transfer heat by two-phase heat transfer devices that utilize the evaporation and condensation of a working fluid, which circulates due to the capillary force developed in a fine porous wick (Ku, 1999). The advantages of LHPs are best manifested at large capacities and heat-transfer distances. Furthermore, LHPs are particularly suitable when it is necessary to ensure efficient transfer at any orientation of the gravity field (Maydanik, 2005).

Since its invention, in 1972 the technology has been significantly improved, and several devices have been significantly developed in the last decades, and at present LHPs are successfully used in a number of space missions (Maydanik, 2005).

LHP systems are mainly auto-controlled, as the maintenance of the temperature close to a certain level is realized automatically without any active external action, but in certain cases the control of the LHP temperature is necessary (Maydanik et al., 1994).

The LHP principle allows creating ramified heat-transfer devices including different numbers of evaporators and condensers situated at different orientations, making themselves particularly suitable for thermoregulation systems of spacecraft, reducing mass, and increasing compactness (Maydanik, 2003).

For the lowest temperatures' applications, Cryogenic Loop Heat Pipes (CLHPs) have been developed (Bai et al., 2012).

**Figure 9:**
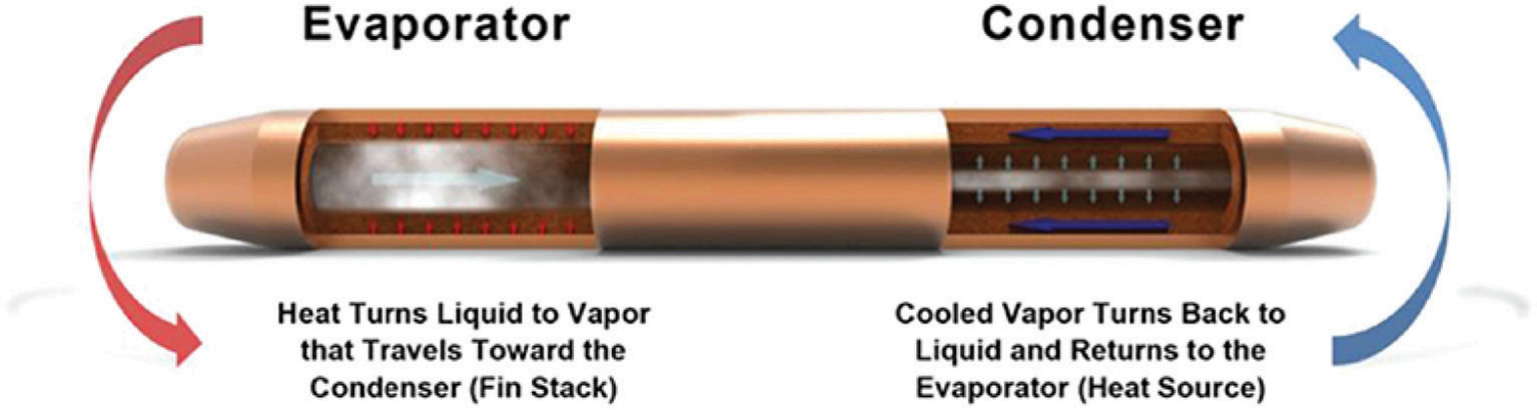
Heat pipe (Source: Elnaggar and Edwan, 2016 in Electronics Cooling, S. M. Sohel Murshed (ed.) DOI: 10.5772/62279)[IntechOpen. CC BY 3.0][Also available from: https://celsiainc.com/heat-pipe-and-vapor-chamber-technology-overview/].

A rendered image of a Heat Pipe is shown in Figure 9.

A different version of LHPs are variable conductance heat pipes (VCHPs), which, for spacecraft thermal control typically have a cold-biased reservoir for a Non-Condensable Gas (NCG) at the end of the condenser. During operation, electrical heat is supplied to the reservoir to provide ±1–2^*o*^*C* temperature control over widely varying powers and sink temperatures. A second application for VCHPs is as a variable thermal link (Anderson et al., 2012).

#### 3.4.2. Fluid loops

A Mechanical Pumped Fluid Loop (MPFL) is a single-phase system that circulates a working fluid via a tubing. As shown in Figure 8B heat will be dissipated to the payload heat exchanger. While the pump provides an almost constant fluid flow, the valve regulates the flow through the radiator heat exchanger. The radiator heat exchanger transfers heat dissipation from the fluid to the external environment. The MPFL is one of the technologies that has enormous potential to meet the demands of future spacecraft thermal control. It is used to transmit a large amount of heat between two regions separated by large distances (Kumar Rai et al., 2015). The working fluid does not undergo any phase change as it flows through various components.

#### 3.4.3. Water sublimators

Those spacecraft working in warm environments can use water sublimators (Tongue and Dingell, 1999), which offer simplicity, reliability, small volume, high efficiency, and excellent work performance in zero gravity (Wang et al., 2014). In general, sublimators are very efficient evaporative heat rejection devices, self-controlling, and without moving parts.

In sublimation mode water freezes in the pores of the plate and heat is removed from the system by sublimation to the vacuum of space. Ice will generate some heat as it freezes from liquid to solid form due to the heat of fusion. Water has an unusually high latent heat of evaporation/sublimation which is enough compensate the heat generated upon fusion (freezing), as well as any heat that might be generated by friction as the water moves through the plate.

A novel approach would be as a supplemental heat rejection device, SHReD. This operational scenario would have the sublimator operating at a cyclical transient heat load with a possible design point for a maximum steady state heat load. A risk that needed to be mitigated for this operational scenario was the reduced utilization of the sublimator when used as a transient heat rejection device. Through this rigorous test program it was concluded that a constant feedwater supply to the sublimator was advantageous over a controlled feedwater supply for cyclical transient heat loads. It should be stressed though that the utilization of the sublimator for constant feedwater supply was still considerably less than that of its steady state utilization (Leimkuehler et al., 2010).

#### 3.4.4. Pulsating heat pipes

Pulsating heat pipes (PHPs), or oscillating heat pipes (OHPs) are one of the latest type of highly efficient heat transfer systems.

As shown in Figure 8C a PHP is a capillary tube bent into turns and filled with a working fluid, which distributes itself in the form of liquid-vapor plugs and slugs. The PHP tube configuration can be arranged in an open-loop or closed-loop design. In the closed-loop design the tube has an end-to-end connection. On the one end of the tube bundle aggregate heat and transfer it to the other end by a pulsating operation of the liquid-vapor system.

The state-of-the-art of experimental investigations on PHPs are mainly focused on the applications of nanofluids and other functional fluids, aiming at enhancing the heat transfer performance of the PHPs (Tang et al., 2013).

**Figure 10:**
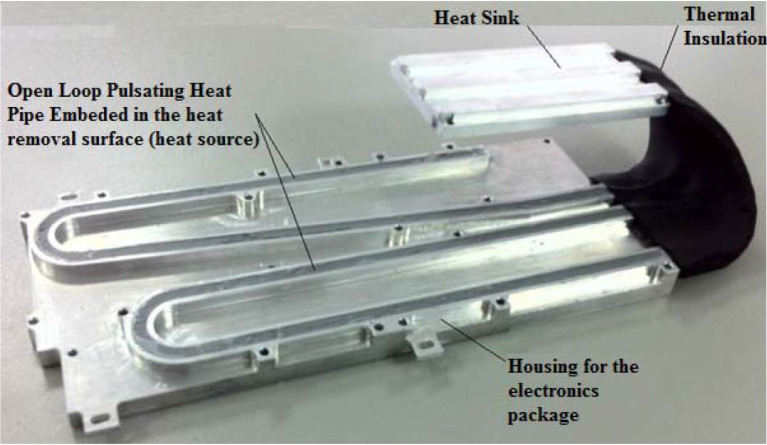
Pulsating heat pipe (Source: Riehl, 2016, doi: 10.4172/2168-9873.1000214) [CC BY].

Figure 10 shows an open loop pulsating heat pipe, where the ends are not connected (Riehl, 2016).

#### 3.4.5. Self-rewetting fluids

The most thermal transfer types work with fluids. The used fluids mainly decrease in the surface tension with increasing temperature. The amount of heat exchange that, e.g., a heat pipe can handle is related to the ability of the condensate liquid layer to counter flow the vaporization process. For wicked heat pipes the capillary pressure due to the wick structure is responsible for stable working fluid circulation and sets an operational limit with respect to the total pressure drop. When this capillary pressure is not adequate to support the flow of liquid from the condenser to the evaporator, the hat pipe is said to have reached his capillarity limit and a dry-out of the evaporator occurs.

Some studies have been conducted on thermal management device, actually wickless heat pipes, with using the so-called “self-rewetting fluids” (dilute aqueous solutions of high carbon alcohols) as a working fluid. Most of liquids show a decrease in the surface tension with increasing temperature, while self-rewetting fluids show exceptionally an increase in the surface tension with increasing temperature: it allows for a spontaneous liquid supply to hotter interface by the thermocapillary flow. When liquid/vapor phase change takes place, furthermore, additional Marangoni effect due to concentration gradient by the preferential evaporation of alcohol-rich composition in the aqueous solutions is induced (Abe et al., 2005).

#### 3.4.6. Hybrid single-phase, two-phase, and heat pump thermal control system

The goal of a hybrid system is to combine at least two technologies in one system. Such a system shall help to solve diverse thermal requirements of different space missions. The Hybrid Thermal Control System (H-TCS) is designed to accommodate three different operational modes: a mechanically single-phase loop for low heat loads, a two-phase loop for high heat loads and a heat pump for hot environments. This system is examined within Orion Multi-Purpose Crew Vehicle (MPCV).

In the single-phase mode, fluid pressure is higher than saturation pressure, and the temperature increase by up to 5 and 40°C as the fluid exits the cabin and avionics heat exchangers, respectively. In the two-phase mode, the working fluid maintains relatively constant saturation temperature and pressure as it absorbs the heat in both the cabin and avionics heat exchangers by changing phase. In the heat pump mode, the same evaporators are used to extract the heat from the cabin and avionics (Lee and Lee, 2016).

## 4. Recommendations

This section aims to propose solutions to be used for electrical power, data and thermal transfer, and mechanical latching, suitable for space and planetary applications, on the basis on the evaluation proposed in the previous section. Table 7 shows the technology readiness level (TRL) of the transfer types described in section 3.

**Table 7:** TRL levels of existing interfaces.

**Type**	**TRL**	**Remarks**	**For orbit**	**For planets**
**MECHANICAL**				
Hook	7–9		Suitable	Suitable
Rotational	7–9	Already developed	Suitable	Suitable
Clamp	7–9	prototyped, tested	Suitable	Limited
Carabiner	7–9	and flown into space	Suitable	Limited
**ELECTRICAL**				
Pins	7–9	Already developed	Limited	Limited
Tab	7–9	prototyped, tested	Suitable	Suitable
Slip ring	7–9	and flown into space	Suitable	Limited
Wireless	3–4	preliminary studies only	Suitable	Suitable
**DATA**				
Milbus	7–9		Suitable	Suitable
CANbus	7–9		Suitable	Suitable
SpaceWire	7–9	Already developed	Suitable	Suitable
Standardized serial interface	7–9	prototyped, tested	Suitable	Suitable
Firewire	7–9	and flown into space	Suitable	Suitable
Time Triggered Ethernet	7–9		Suitable	Suitable
Time-Triggered Bus	7–9	Aircraft only	Limited suitable	Limited suitable
**THERMAL**				
Heat pipes	7–9	Already developed	Suitable	Suitable
Fluid loops	7–9	prototyped, tested	Suitable	
Water sublimators	7–9	and flown into space		Suitable
Pulsating heat pipes	6–7	Never in space	Suitable	Suitable
Self-rewetting fluids	5–7	Parabolic flights only		

In order to assess the suitability or each transfer type to the two environments, the influences of space and planetary conditions have been considered, such as temperature range, effects on materials, atomic oxygen erosion, radiation, single event effects (SEEs), gravity and magnetism, atmosphere, high vacuum, and contamination (dust particles and space debris) (Ley et al., 2009).

### 4.1. Recommended latching methods

Depending on required application for mechanical latch types, all mentioned latching mechanism in section 3.1. can be used. Hook, rotational lock, or clamp type latches do not require physical translated force against the target interface to latch, and offer the advantages of androgynous design.

The hook, rotational lock, and clamp mechanism cover passive retention, fail-safeness is mainly provided by the rotational lock and the clamp methods, in contrast the clamp and carabiner mechanism offer significant misalignment correction. Thus, it is meaningful to evaluate the requested features of a latch mechanism for orbital or planetary use.

An example for an androgynous, self-correcting clamp is the geometry from SINGO (Shen et al., 2009) and iBoss (Goeller et al., 2016).

### 4.2. Recommended power transfer methods

Power transfer interface aims compactness and robust design, low weight, protection against short circuit, multiple usage, and space environment robustness. Due to its high tolerance for dust particles and compounded by the popularity of the method in existing interface designs, spring loaded contacts (tabs) are recommended as electrical interface (Dettmann et al., 2011).

The popularity and continuing extensive use of 100 V/100 A/10 kW platforms, leads to the conclusion that these should be the minimum requirement benchmark for any electrical interface design.

### 4.3. Recommended data standards

Main requirements for the data interface are compactness and robustness, low mass, multiple usage, compliance with space environment conditions, and the highest possible data rate (at least 100 Mbit/s).

In terms of the physical interface, recommended features are: the use of differential-driven signaling (e.g., LVDS) for high data rate, synchronous, and clocked (or self-clocking) operation to prevent timing variations, the use of shielded differential twisted pair data lines for noise rejection, the use of redundant pairs (at least two) in a given data link in case of a bad contact, full-duplex operation, connectors that ensure correct polarity and positive contact locking against vibration, connectors made of materials that will not corrode or accumulate nonconductive layers in planetary or vacuum environments, voltage high enough to overcome radiation and static buildup effects but not high enough to be hazardous, external contacts and wiring electrically isolated at the transceiver by means of optical or pulse transformer coupling to ensure protection against static discharge.

Many industry-standard communications methods may not be suitable on the basis of the above requirements. Standardized serial interfaces such as RS-422 and RS-485 are electrically outdated and are typically limited in data rate well below 100 Mbit/s and require higher voltages than standards such as LVDS and power in the range of 350 mW, although some updated hardware for these standards exists. The ECSS standard ECSS-E-ST-50-13C describes the use of MIL-STD-1553B Milbus on board spacecraft, but it is also limited in maximum data rate below 100 Mbit/s due to the use of time-division multiplexing and Manchester encoding and minimum (secondary) power consumption is 2 W. ECSS-E-ST-50-15C describes the use of CAN bus in space hardware, with CANOpen chosen as the standard protocol layer for ESA missions (Taylor et al., 2015).

There is current development toward low-power rad-hard ISO 11898-2 CAN transceiver hardware and CAN can be implemented over twisted pairs also. The main limitation of CAN is bit rate, with a maximum of 3.7 Mbit/s in the CAN FD standard, which prevents it from reaching the desired rate of 100 Mbit/s. SAFEBus is limited to 60 MB/s, Time-Triggered CAN to 1MB/s, and other standards like TTA are implemented over an Ethernet physical layer if high speed is desired (Rushby and Miner, 2003).

The remaining physical layer standards are very similar in design. Ethernet, which is isolated twisted-pair based, most accessible and common to many bus protocols including Time-Triggered Ethernet and EtherCAT provides up to 1 Gbit/s depending on underlying hardware. Firewire is very flexible in implementation, also usually twisted-pair based, and utilized in aerospace using SAE aerospace standard AS5643 which adds many features including a looped topology, transformer isolation (like Ethernet), time synchronization, and multiple-path redundancy. SpaceWire is typically limited by hardware design to 400 MBit/s (50 MB/s), but the underlying LVDS standard can perform much faster with up to 3 Gbit/s possible in concept on terrestrial hardware. It may be advisable to make use of the LVDS SpaceWire interface standard, but without the requirement that only SpaceWire protocol be transmitted over it, and with the addition of robust isolation.

To accommodate the widest possible number of future usages, it is suggested that the best common features of these standards be used to match up with the recommended features given above. With respect to the physical layer alone, all of these recommended standards include differential signaling, transformer or similar isolation methods, and full-duplex operation. The use of redundant pairs and a separate clock line are desirable features to add as a capability to the physical interface (though self-clocking is possible if suitably resilient and if maximum bit rate is not heavily affected). Ethernet standards provide the most appropriate basis for high-speed signaling, but features such as redundant operation and a physical clock may be desirable. If at all possible, the hardware standard for interfacing should be designed to satisfy the most stringent of requirements so as to allow mixed or common use of Time-Triggered Ethernet/EtherCAT, LVDS/fast SpaceWire, and in an extended case a modified Firewire AS5643 implementation.

In conclusion, the best data protocol seems to be the LVDS SpaceWire interface standard (Parkes, 2010), but without the requirement that only SpaceWire protocol be transmitted over it, and with the addition of robust isolation.

### 4.4. Recommended heat transfer methods

Thermal transfer between spacecraft modules, robot manipulators, and payloads in space are not a well traversed knowledge area, thus further detailed research by experts is recommended to build confidence in this area. A thermal interface in space needs to be compact and robust, have low weight and complexity, allow multiple usage, be an active system. Recommendations for satisfying these requirements are:

- Heat pipes represent the most reliable thermal interface, already applied in several spacecrafts;
- Fluid loop is the technology capable of carrying on the largest amount of heat at the longest distance. Most critical issues is that leakage may occur due to the void environment.
- Self-rewetting fluid technology appears to be the most promising one. Wickless heat pipe shows better thermal resistance and higher dry out limit than ordinary heat pipe, but a few issues have still to be addressed.

### 4.5. Open niches and novelty area

The novel areas that can help differentiate a design from its peers, determined by an absence in the literature or a notable lack of recent improvement, have been here listed.

#### 4.5.1. Total self-alignment

The free-lying nature of spacecraft means that often the orientation of a spacecraft cannot be guaranteed, or requires expensive correction. In the context of orbital servicing there could also be a rogue target craft with no ability to correct itself (Nanjangud et al., 2018). In this case, aligning the interfaces will be a difficult and timely task. By simplifying the interface approximation process so that in any angle of approach the system will self-align to the required angle would represent a high value and step forward from the state of the art.

#### 4.5.2. Pseudo-infinite orientation

An alternative solution to the orientation problem is to design an interface that would mate with the counterpart in a high number of possible orientations. This will involve designing the interface with circular fixtures for power and data, and possibly a central exchange for thermal. An example of how this kind of design might manifest is the Phoenix Satlet module design. While there are a countable amount of final latched orientations, one can count it as pseudo-infinite as angle between each acceptable state is negligible. On a true pseudo-infinite platform, the interface angle between any two modules could be of any angle (with a resolution of 1° or less), opening up options for more complex arrangements, and eliminating the need for re-alignment.

#### 4.5.3. No-push mating system

On the same subject of spacecraft correction; pushing two interfaces to activate the latch is an expensive task. This is usually solved with the inclusion of an independent manipulator grappling the target. By creating an interface geometry that either pulls the target toward the chaser, or latches with torque without pushing the target away, it would be possible to effectively capture free floating craft with little risk of it accelerating away. This is also vital in the scenario of a defunct, rogue craft as it may not have any means of correcting itself if the latching process transfers translational force.

#### 4.5.4. Hybrid technical interface

Docking interfaces have to cope with different influences in the fields of application. An interface, which is going to work in orbital and planetary environment, has to cover different requirements, e.g., the ability to work in different temperatures. Working in a planetary environment needs other solutions for mechanical, data, power, and thermal transfer types than in an orbital environment. This implies that an interface could need two different transfer types for data, thermal, mechanical, and electrical parts. Here a hybrid technical transfer type may be a solution. In case of the different temperature ranges the design of thermal transfer interface could be designed for hybrid use. A possible way is a matched hybrid thermal control system (Lee and Lee, 2016).

## 5. Conclusion and outlook

This review aimed to present the state-of-the-art and the future perspective of robotic space interfaces, with focus on thermal, data, electrical, and mechanical functionalities. The main conclusions are here summarized:

1. Many existing interface designs target small modular robots, but the design principle can be up scaled:
  - The iSSI is the closest existing prototype that integrates all the four main functionalities described here;
  - Rotational symmetry, internal redundancy, and androgynous connection are common and basic requirements;
  - Additional design effectiveness is achieved with particle mitigation, 6-DoF misalignment tolerance, and fail-safe docking and undocking;
2. Latching methods consist of four archetypes; hook, clamp, carabiner, and rotational lock;
3. Electrical power transfer methods included tabs, slip rings, pin arrangements, and even wireless:
  - Scoop proof and spring loaded tab contacts are recommended physical means of power transfer;
  - 100 V bus minimum requirements are recommended as a benchmark;
  - Slip rings can also be taken if a pseudo-infinite orientation design is pursued;
4. Data transfer protocols ranged from CANbus to SpaceWire and Firewire:
  - The use of redundant twisted pairs and fullduplex is recommended;
5. Thermal exchange methods are rarely applied in such a way, but usually took the form of heat pipes or fluid loops:
  - Heat pipes represent the simplest method, but fluid loops/rewetting fluids have the most potential;
  - Only one design with integrated thermal interface has been found, and it still needs further development.

In conclusion, the path toward a development of a standard space interface with integrated multiple functionalities, has still a long way to go, but the current developments are moving in the right direction.

## Author contributions

X-TY is the coordinator of the unit that developed this literature review. WB provided help with writing, literature review and created sketches of the different transfer types. RP is the main contributor, and in charge of writing the paper. CM helped with literature review and provided most of the figures. YL helped with the literature review. SB provided input on the review and helped with the writing. FK is the coordinator of the German group an helped with writing.

### Conflict of interest statement

The authors declare that the research was conducted in the absence of any commercial or financial relationships that could be construed as a potential conflict of interest. The reviewer, DF, and handling Editor declared their shared affiliation.
